# The gut-bone axis: impact of diet on gut microbiome and osteoporosis

**DOI:** 10.1038/s41413-026-00550-4

**Published:** 2026-06-04

**Authors:** Yue Wang, Miao Xu, Qinzuo Dong, Xuan Lin, Qiang Zhang, Hua Shi, Xiaoning Lin, Huan Liu, Min Yu, Jianlin Shen

**Affiliations:** 1 Central Laboratory, Affiliated Hospital of Putian University, School of Basic Medicine, Putian University, Putian, Fujian China; 2 Key Laboratory of Translational Tumor Medicine in Fujian Province, Putian University, Putian, Fujian China; 3 School of Basic Medicine, Fujian Medical University, Fuzhou, Fujian China; 4 First Clinical Medical College, Yunnan University of Chinese Medicine, Kunming, Yunnan China; 5 Second Clinical Medical College, Yunnan University of Chinese Medicine, Kunming, Yunnan China; 6 Department of Orthopedics, Affiliated Hospital of Putian University, Putian, Fujian China; 7 Department of Orthopaedics, The Affiliated Traditional Chinese Medicine Hospital, Southwest Medical University, Luzhou, Sichuan China; 8 Department of General Surgery, Chongqing General Hospital, Chongqing University, Chongqing, China

**Keywords:** Metabolic bone disease, Endocrine system and metabolic diseases, Endocrine system and metabolic diseases

## Abstract

Osteoporosis, a global health challenge characterized by reduced bone mineral density (BMD) and increased fracture risk, is closely linked to interactions between dietary patterns and gut microbiota (GM). Recent advances in GM research highlight the gut-bone axis as a pivotal focus in osteoporosis studies, revealing that variations in dietary habits and nutrient intake differentially modulate GM composition and metabolic activity, thereby influencing skeletal health. This review synthesizes current evidence on the interplay between dietary practices, GM, and bone health, aiming to identify preventive and therapeutic strategies for osteoporosis management. Collectively, these findings underscore the potential of dietary interventions targeting GM modulation as a novel therapeutic approach, advocating for personalized nutritional strategies to improve skeletal health in high-risk populations.

## Introduction

Osteoporosis is a systemic skeletal disorder characterized by reduced bone mineral density (BMD), deterioration of bone microarchitecture, and diminished bone strength, which collectively culminate in an elevated fracture risk.^1^ With aging populations and increased life expectancy globally, this often-asymptomatic condition has become a major public health burden, frequently remaining undiagnosed until fracture occurrence. Such fractures substantially reduce quality of life, increase disability, elevate healthcare costs, and may cause premature mortality.^2^

Bone tissue undergoes continuous remodeling, regulated by genetic predisposition, pharmacological agents (e.g., glucocorticoids), lifestyle factors, and dietary intake; an imbalance favoring bone resorption drives osteoporosis pathogenesis.^3–6^ Although the roles of specific nutrients in bone health are well-established,^7,8^ comprehensive analysis of dietary patterns is essential due to the synergistic effects of food combinations. Epidemiological and experimental evidence indicates that Western dietary patterns promote osteoporosis development,^9,10^ whereas Mediterranean diet correlate with improved bone health and reduced fracture risk.^11,12^ While vegetarian diets, particularly vegan regimens, are often associated with lower BMD,^13^ strategic nutrient fortification or supplementation can mitigate fracture risk.^14^

Critically, the gut microbiota (GM) plays a central role in regulating bone density, with dysbiosis directly implicated in osteoporosis pathogenesis.^15–17^ For example, germ-free mice exhibit higher bone mass than specific pathogen-free counterparts, whereas colonization with microbiota from osteoporotic models induces bone loss.^18–20^ Dietary patterns directly shape GM composition: pro-inflammatory Western diets enrich Proteobacteria while depleting short-chain fatty acid-producing bacteria,^21^ whereas plant-rich diets promote beneficial taxa such as *Ruminococcus* and *Roseburia*.^22^ Furthermore, emerging evidence confirms that dietary modulation of the GM impacts bone health; higher fiber intake correlates with reduced osteoporosis prevalence in postmenopausal women.^23,24^ This review synthesizes current knowledge on the diet-GM-bone interplay, providing a foundation for microbiota-targeted nutritional strategies to prevent and manage osteoporosis.

## Osteoporosis as a disease of the gut-bone axis

Osteoporosis is a systemic skeletal disorder characterized by low bone density, deterioration of bone tissue, disrupted bone microarchitecture, compromised bone strength, and an increased risk of fracture.^8^ Its pathogenesis involves dysregulated bone remodeling, commonly seen in elderly individuals and postmenopausal women.^25^ Central to this process is an imbalance between bone-forming osteoblasts and bone-resorbing osteoclasts; when osteoclast-mediated resorption exceeds osteoblast-mediated formation, bone mass and structural integrity decline.^26^ Although traditionally classified as an endocrine disorder due to estrogen deficiency, osteoporosis is increasingly recognized as a chronic, inflammation-driven condition with significant immune involvement.^27^

Concurrently, growing understanding of the gut-bone axis supports a paradigm shift toward a multifactorial pathogenesis, highlighting the central role of intestinal microbiota in regulating skeletal homeostasis. First, epidemiological studies have shown that the global increase in the incidence of osteoporosis is related to multiple factors, among which the Western diet is an important factor.^28^ Second, germ-free mice exhibit higher bone mass than conventionally colonized counterparts, despite comparable hormonal environments.^29^ Third, fecal microbiota transplantation (FMT) from healthy mice into the ovariectomized (OVX) mice model results in decreased bone loss within eight weeks.^19^ Collectively, this evidence positions the GM as a critical environmental modulator of bone health.

The molecular mechanisms bridging gut and bone are multifaceted. Estrogen deficiency, a key driver of postmenopausal osteoporosis (PMOP), triggers systemic immune activation. This expands populations of T cells and macrophages that secrete pro-inflammatory cytokines such as interleukin-1β (IL-1β), IL-6, and tumor necrosis factor-alpha (TNF-α).^30^ These cytokines enhance osteoclastogenesis by stimulating RANKL expression in osteoblasts and T cells, and by activating NF-κB and MAPK signaling in osteoclast precursors. IL-1β further amplifies bone resorption by suppressing osteoprotegerin (OPG), a key inhibitor of RANKL.^31^ Meta-analyses confirm that serum IL-6 and TNF-α levels are significantly elevated in PMOP.^32^ Moreover, activation of the NLRP3 inflammasome exacerbates bone loss by promoting the maturation and release of IL-1β and IL-18, which disrupt osteoblast differentiation and shift mesenchymal stem cell lineage commitment toward adipogenesis. Concurrently, oxidative stress and reactive oxygen species contribute to osteoblast apoptosis and further inhibit bone formation.^33–35^ Thus, a chronic low-grade inflammatory state forms a core pathological bridge between systemic triggers (like estrogen loss) and local bone catabolism.

**Fig. 1:**
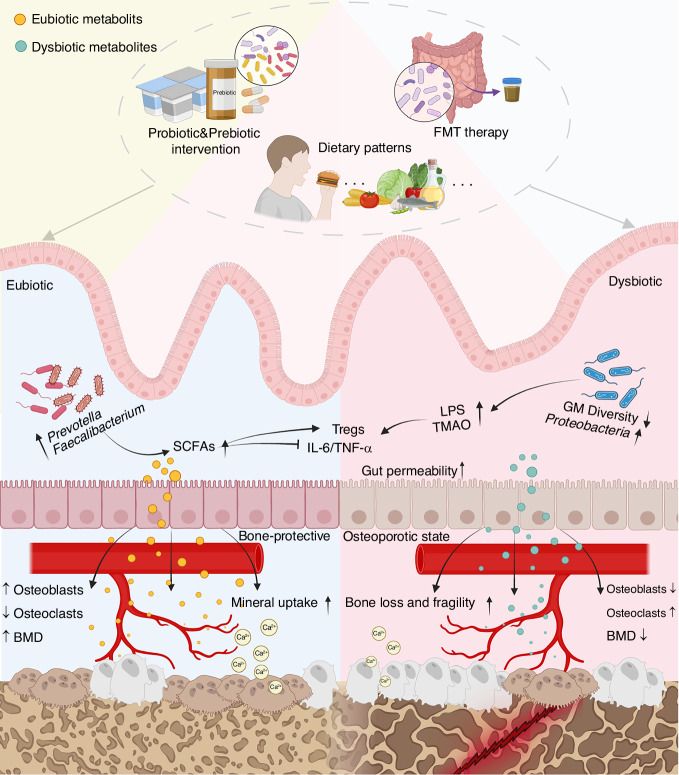
Mechanistic map of the Diet-GM–Bone Axis in osteoporosis. This schematic illustrates the impact of GM on bone health through bidirectional modulation by dietary patterns, probiotic/prebiotic interventions, and FMT. Left panel (Eubiotic State): A healthy gut environment is characterized by beneficial bacteria such as *Prevotella* and *Faecalibacterium*. These beneficial bacteria produce SCFAs, which inhibit pro-inflammatory cytokines (IL-6/TNF-α) and promote Tregs. Eubiotic metabolites enhance mineral (Ca^2+^) uptake, promote osteoblast activity, and suppress osteoclast differentiation, collectively leading to increased BMD and bone protection. Right panel (Dysbiotic State): Gut dysbiosis is marked by reduced GM diversity and increased harmful bacteria (e.g. *Proteobacteria*). This state leads to increased intestinal permeability and the accumulation of harmful metabolites such as LPS and TMAO. Upon entering the systemic circulation, these dysbiotic metabolites suppress osteoblast function and activate osteoclasts, ultimately resulting in bone loss, decreased BMD, and increased bone fragility (osteoporotic state). Key: Yellow dots = Eubiotic Metabolites; Blue dots = Dysbiotic Metabolites. SCFAs short - chain fatty acids, Tregs regulatory T cells, TNF-α tumor necrosis factor-α, IL-6 interleukin-6, BMD bone mineral density, FMT fecal microbiota transplantation, LPS lipopolysaccharide, TMAO trimethylamine N-oxide, GM gut microbiota. Created with BioRender.com

Beyond these classical endocrine-immune pathways, the GM critically regulates bone metabolism through the production of bioactive metabolites and direct immunomodulation, forming the functional basis of the gut-bone axis^36,37^ (Fig. 1). This axis functions via two core, opposing mechanisms, with the outcome modulated by the type and implementation of specific interventions, such as dietary modifications, probiotic/prebiotic use, and FMT. The protective (eubiotic) pathway is promoted through beneficial dietary patterns and positive microbial interventions, which enrich beneficial microbiota in the gut. For instance, diets such as the Mediterranean or plant-based diets stimulate the growth of fiber-fermenting bacteria, including *Faecalibacterium* and *Prevotella*.^38,39^ These bacteria produce short-chain fatty acids (SCFAs) including butyrate.^40^ SCFAs serve as pivotal signaling molecules that: (i) directly inhibit osteoclast activity;^41,42^ (ii) promote the expansion and function of regulatory T cells (Tregs);^40^ and (iii) suppress pro-inflammatory cytokines like IL-6 and TNF-α.^41^ Additionally, bacterial components such as extracellular vesicles from *Akkermansia muciniphila* can migrate to bone and directly stimulate osteoblast activity.^43^ Collectively, this SCFA-driven, anti-inflammatory milieu promotes osteoblast function and mineral uptake while inhibiting osteoclastogenesis, culminating in enhanced BMD and a bone-protective state.

Conversely, the destructive (dysbiotic) pathway is triggered by long-term consumption of a Western diet (high in fat/sugar, low in fiber) and negative microbial interventions, which induces GM dysbiosis.^44^ This dysbiotic state is characterized by reduced microbial diversity, depletion of SCFA-producing bacteria, and expansion of pro-inflammatory taxa such as *Proteobacteria*.^33,44^ This shift entails two major deleterious consequences. First, the increase in Gram-negative *Proteobacteria* elevates production of lipopolysaccharide (LPS). Diet-induced impairment of the intestinal barrier facilitates LPS translocation into circulation, triggering systemic inflammation that fuels osteoclastogenesis and bone loss.^45,46^ Second, dysbiotic bacteria such as *Proteobacteria* and *Shigella* convert dietary nutrients into trimethylamine (TMA),^47^ which is hepatic converted to trimethylamine N-oxide (TMAO). Elevated plasma TMAO correlates negatively with BMD and positively with TNF-α levels, directly linking it to inflammation-driven osteoporosis.^47–49^ Thus, the dysbiotic pathway is marked by dominance of harmful metabolites (LPS, TMAO), elevated pro-inflammatory cytokines (IL-6/TNF-α), increased osteoclast activity, and consequent bone loss and fragility.

In summary, osteoporosis is reconceptualized as a disorder of the gut-bone axis, where dietary patterns dictate GM ecology, which in turn modulates systemic immune and metabolic signals that directly control bone remodeling. These mechanistic insights underscore the potential of targeted interventions—such as probiotics, prebiotics, synbiotics, and FMT—to modulate the GM and thereby influence bone health. This framework establishes the diet–microbiota–bone axis as a novel and actionable target for the prevention and management of osteoporosis.

## Gut-bone axis mechanisms

### Gut microbiota

Growing evidence implicates gut microbiota (GM) dysbiosis in osteoporosis pathogenesis. Patients with osteoporosis and OVX mice consistently exhibit reduced microbial diversity and altered composition, characterized by depletion of beneficial taxa (e.g., *Lactobacillus*, butyrate-producers).^50–55^ Dietary patterns significantly influence GM shifts: For instance, postmenopausal osteoporotic women consume less yogurt and fruit but more red meat and alcohol than healthy controls, correlating with decreased *Streptococcus* and increased pro-inflammatory taxa (e.g., *Nocardiaceae*, *Rhodococcus*).^56^

Large-scale microbiome profiling studies further reinforce associations between GM dysbiosis and low BMD. Osteoporotic individuals show reduced microbial richness and genus-level alterations, including enrichment of *Allisonella*, *Klebsiella*, and *Megasphaera* (positively correlated with bone turnover markers),^36,57^ along with elevated *Firmicutes/Bacteroidetes* ratios. Depletion of anti-inflammatory genera (e.g., *Lachnospira*, *Collinsella*, *Veillonellaceae*) and enrichment of *Actinobacillus*, *Oscillospira*, and *Phascolarctobacterium* are also observed.^58–60^ Animal models further link *Helicobacter* enrichment and decreased *Firmicutes/Bacteroidetes* ratios to bone loss,^61^ while Mendelian randomization analysis associates reduced *O_Burkholderiales* and increased *g_Ruminococcus* with OVX-induced osteoporosis.^62^ Notably, low *Prevotella* abundance in PMOP patients is functionally significant, as orally given *Prevotella* prevents bone loss in OVX mice.^63,64^

Experimental interventions highlight GM’s therapeutic potential for bone health. Probiotics (e.g., *Lactobacillus brevis* AR281, *L. rhamnosus and Bacillus coagulans*) improve BMD by suppressing RANKL/OPG imbalance and inflammatory cytokines via TNF–TRAF6–NF-κB–NFATc1 pathways.^65,66^ Additionally, specific strains (*Akkermansia*, *Bifidobacterium*, *Lactococcus lactis*) enhance gut barrier integrity and inhibit osteoclastogenesis,^67–70^ and fungal supplements (e.g., *Isaria felina, Cystofilobasidium*) correct Th17/Treg imbalance while reducing bone-resorptive genera (*Bacteroides*, *Ruminococcus*).^71,72^ Notably, eukaryotic viruses may indirectly exacerbate osteoporosis by inducing inflammation-driven bone resorption.^73^ Dietary modulation also impacts GM-bone crosstalk: Adherence to Mediterranean diet elevates *Prevotella*-to-*Bacteroides* ratios and diversity.^74,75^ while Mediterranean diet-fed mice show increased *Lactobacillus*/*Faecalibacterium* and decreased *Ruminococcus* versus Western diet-fed mice.^76,77^

Metabolomic studies reveal that SCFA-producing taxa (*Subdoligranulum*, *Muribaculaceae*, *Alistipes*) correlate with BMD in PMOP.^78^ Notably, *Lactobacillus*, *Akkermansia*, *Prevotella*, and *Butyricicoccus* are consistently depleted in low bone mass and PMOP cohorts.^67,79^ Key dysbiosis patterns are summarized in Table 1. It should be noted that microbial functions are often strain- and context-dependent; the associations listed in the table represent general trends rather than definitive functional assignments. A prime example is SCFAs, which can exhibit biphasic or context-dependent effects on bone. For instance, propionate derived from *Veillonellaceae* is generally anti-inflammatory, yet its overproduction may contribute to pathological states.^80–82^ Butyrate demonstrates a clear dose-dependent duality: at lower levels, it promotes bone formation via Wnt signaling and Treg activation,^83,84^ whereas at higher levels, it may stimulate pro-inflammatory cytokines and osteoclastogenesis, particularly in an inflammatory milieu.^83^ These nuances highlight the complexity of translating microbial signatures into mechanistic insights. Although specific bacterial alterations vary across studies—potentially due to methodological or cohort differences—the overall link between GM dysbiosis and osteoporosis is well-established.

**Table 1:** Dysbiosis associated with osteoporosis

Phylum	Family	Genus/Species	Microbiota changes	Associated effects
Actinobacteria	*Coriobacteriaceae*	*Collinsella*^59^	**↓**	Negatively correlated with osteoporosis
	*Bifidobacteriaceae*	*Bifidobacterium* (e.g., *B. longum*)^68,69^	**↑**	Increased vitamin D metabolites, reduced bone loss
Firmicutes	*Lactobacillaceae*	*Lactobacillus* (e.g., *L. fermentum*, *L. salivarius*, *L. brevis*, *L. paracasei*)^52,53,55,56,66,79^	**↑**	Supplementation increases bone volume and BMD
	*Bacillaceae*	*Bacillus* (e.g., *B. coagulans*)^65^	**↑**	Improved BMD and microarchitecture via immune modulation
	*Lactococcaceae*	*Lactococcus lactis*^68^	**↑**	NF-κB inhibition, improved microbiota balance
	*Lachnospiraceae*	*Lachnospira*,^60,336,337^ *Roseburia*,^58,338^ *Blautia*^36^	**↓**	Produces various SCFAs (e.g., butyrate, propionate, acetate) that may influence bone metabolism
	*Ruminococcaceae*	*Ruminococcus*,^36,72^ *Subdoligranulum*^78^	–	Affects BMD by regulating SCFA- production
	*Veillonellaceae*	*Veillonella*,^59,82,339^ *Megasphaera*^57^	–	Produces multiple SCFAs including propionate and acetate, with anti-inflammatory properties and context-dependent effects on bone remodeling.
Bacteroidetes	*Bacteroidaceae*	*Bacteroides*^72^	–	Regulates intestinal flora diversity
	*Parabacteroidaceae*	*Parabacteroides*^36^	–	Anti-inflammatory properties
	*Prevotellaceae*	*Prevotella*^63,64^	**↓**	Potential bone-protective effects in osteoporosis
	*Rikenellaceae*	*Alistipes*^57^	**↑**	Associated with anti-inflammatory effects
Proteobacteria	*Enterobacteriaceae*	*Klebsiella*,^57^ *E. coli–Shigella*^67^	**↑**	Inflammatory dysbiosis, increased bone turnover
	*Helicobacteraceae*	*Helicobacter*^61^	**↑**	Potentially related to osteoporosis as a risk factor
	*Pasteurellaceae*	*Actinobacillus*^59^	↑	Relative abundance is positively correlated with osteoporosis
Other	–	*Allisonella*^57^	**↑**	Positively correlated with serum procollagen type I N propeptide and C-terminal telopeptide of type I collagen
Fungi	–	*Isaria felina*^72^	–	Reversed osteoporosis via Th17/Treg immune modulation
	–	*Cystofilobasidium*^71^	**↑**	Negatively correlated with BMD
Viruses	–	Gut bacteriophages^73^	**↑**	Viral-induced microbiota disruption, inflammation, exacerbated bone loss

Italic text indicates microbial names; references cited in the table correspond to the reference list at the end of the article

*BMD* bone mineral density, *NF-κB* nuclear factor kappa-light-chain-enhancer of activated B cells, *SCFAs* short-chain fatty acids, *Th17* T helper 17 cell

Collectively, these findings support a functional gut-bone axis wherein microbial dysbiosis perturbs immune regulation, intestinal barrier function, and metabolic signaling to disrupt bone remodeling. Targeting GM represents a promising therapeutic strategy for osteoporosis, particularly in postmenopausal women.

### Intestinal barrier

The intestinal barrier — composed of the mucosal epithelium, mucus layer, GM, and immune cells — plays a pivotal role in maintaining host homeostasis by selectively permitting nutrient absorption while blocking translocation of harmful substances into systemic circulation.^85^ Critically, disruption of this barrier is increasingly recognized as a key factor in osteoporosis development. Among the major contributors to barrier dysfunction, GM dysbiosis and estrogen deficiency stand out. Notably, tryptophan deficiency impairs epithelial integrity, thus promoting intestinal inflammation and bone degradation. Conversely, animal studies demonstrate that supplementation with tryptophan-producing bacteria restores intestinal barrier function and significantly reduces bone loss.^86,87^

Alterations in GM composition are frequently observed during OP progression. For example, fluctuations in the abundance of *Order Burkholderiales* and *Genus Ruminococcus* show strongly correlations with intestinal barrier function indices, suggesting that their dysregulation may contribute to barrier impairment.^62^ Furthermore, FMT from aged osteoporotic rats to healthy recipients has been demonstrated to induce GM dysbiosis and trigger the onset of osteoporosis,^20^ providing direct evidence that microbial imbalance causes barrier dysfunction and bone loss.

Targeting the GM has yielded promising therapeutic outcomes. For instance, administration of *Lactobacillus rhamnosus* GG (LGG) to OVX rats not only promoted osteogenesis but also mitigated inflammation and mucosal injury associated with the intestinal barrier. Additionally, LGG restored the Th17/Treg balance and reversed the estrogen deficiency-induced elevation in the *Firmicutes*/*Bacteroidetes* ratio, thereby improving both intestinal and skeletal health.^88^ Similarly, treatment with the anti-inflammatory bacterium *Bifidobacterium lactis* BL-99 in a murine colitis model significantly upregulated intestinal barrier protein expression and enhanced bone microarchitecture, indicated by increased bone volume fraction, trabecular number, and thickness.^89^ Furthermore, multiple probiotic strains have effectively reduced inflammation-induced bone loss.^64,66,90^

Collectively, these findings demonstrate the beneficial effects of specific microbiota modulation on bone health. Conversely, exposure to pathogen-derived toxins can impair the intestinal barrier, promote inflammation, and elevate the production of pro-inflammatory microbial products such as LPS. This cascade triggers pyroptosis in osteoblasts and inhibits their differentiation, ultimately exacerbating bone deterioration.^91^

### GM-immune-bone axis

The immune system critically regulates bone metabolism, with the GM serving as a key intermediary through its dietary-modulated composition and diversity. GM alterations directly influence systemic inflammation, nutrient metabolism, and immune signaling pathways—core mechanisms governing bone remodeling and osteoporosis susceptibility.^92,93^

Notably, dysbiosis is characterized by an increased *Firmicutes*/*Bacteroidetes* ratio and overrepresentation of pro-inflammatory bacteria such as *Enterobacteriaceae*.^94–96^ These Gram-negative bacteria produce LPS, a potent immunostimulant that activates Toll-like receptor 4, promoting the release of IL-6 and TNF-α. This inflammatory cascade promotes osteoclastogenesis, suppresses osteoblast function, and accelerates bone loss.^93,97,98^

Beyond LPS-mediated inflammation, specific gut microbes—particularly *Ruminococcus gnavus*—contribute to bone deterioration by producing TMA. Hepatic conversion of TMA to TMAO induces systemic inflammation and gut barrier disruption. Elevated serum TMAO levels, observed in aged mice and postmenopausal women with hip fractures, correlate negatively with BMD.^48,99–101^

Conversely, beneficial taxa such as *Prevotella* and *Lactobacillus* play protective roles in skeletal health. *Prevotella* species ferment dietary fibers into SCFAs, predominantly butyrate. Butyrate activates AMP-activated protein kinase (AMPK) in intestinal epithelial cells, thereby strengthening gut barrier function and modulating immune cytokine expression through suppression of IL-1β, IL-6, and TNF-α and promotion of anti-inflammatory factors such as IL-10 and TGF-β.^102–104^ These immunometabolic shifts inhibit osteoclastogenesis and stimulate osteoblast differentiation.

Multiple studies have further demonstrated the osteoprotective effects of *Lactobacillus rhamnosus, L. helveticus, and Faecalibacterium*, which are associated with increased butyrate production, expansion of regulatory T cells, and stimulation of Wnt10b signaling via CD8⁺ T cells in the bone marrow.^105–107^ Additionally, *Lactobacillus* strains enhance vitamin D receptor expression and calcium absorption in intestinal epithelial cells, thereby improving bone mineral availability.^108,109^

Critically, the immune system functions as a key intermediary by translating GM-derived pro-inflammatory signals (e.g., LPS and TMAO) into increased osteoclastogenesis and suppressed bone formation. Simultaneously, disruption of the intestinal barrier facilitates microbial translocation and systemic inflammation, exacerbating skeletal deterioration. Notably, interventions targeting GM—including probiotics, dietary modulation, and metabolite administration—exert osteoprotective effects through microbial balance restoration, intestinal barrier reinforcement, and immune response modulation. Thus, the GM emerges as a novel and multifaceted therapeutic target in the prevention and treatment of osteoporosis, particularly in postmenopausal populations.

## Key microbiota-derived metabolites and micronutrients

### Short-chain fatty acids

Short-chain fatty acids (SCFAs) are metabolites produced when dietary fiber is fermented by gut bacteria, a process that links host nutrition to intestinal homeostasis. These molecules function as critical signaling compounds in the gut–bone axis by modulating intestinal barrier integrity, immune responses, and bone metabolism. The concentration and composition of SCFAs are primarily determined by dietary fiber intake and the abundance of SCFAs-producing bacteria, including *Faecalibacterium prausnitzii*, *Roseburia* and *Bifidobacterium* species.^110,111^

Among SCFAs, butyrate plays a particularly important role in maintaining gut epithelial homeostasis. It serves as the primary energy source for colonocytes and promotes the expression of tight junction proteins,^112^ which enhances intestinal barrier integrity and reduces endotoxin translocation into systemic circulation.^113^ Although other SCFAs are less effective than butyrate in barrier regulation, they still contribute significantly to systemic immune function.^104,114,115^

SCFAs directly influence bone metabolism by modulating osteoclast and osteoblast activity. Specifically, butyrate enhances osteoblast differentiation via activation of the Wnt10b/NF-κB signaling pathways and suppresses osteoclast formation through the regulation of transcription factors.^116^ Acting as a critical link between dietary patterns, GM composition, and bone homeostasis, SCFAs demonstrate beneficial effects on intestinal barrier function, immune regulation, and bone metabolism. Therefore, supplementation with high-fiber diets, prebiotics, and related compounds that promote SCFAs production may represent an effective strategy for preventing and treating osteoporosis.

### Dietary fiber

Vegetarian diets are typically rich in dietary fiber, primarily derived from whole grains, legumes, vegetables, and fruits. Specifically, grain-based foods contribute ~17.8% of total intake, followed by fruits (14.9%), vegetables (13.7%), and legumes (6.3%).^117^ Beyond supporting digestive health, fiber exerts systemic benefits through GM modulation, including enhanced antioxidant enzyme activity, reduced erythrocyte lipid peroxidation, and regulated hepatic detoxification pathways.^117^ Crucially, its role in gut–bone axis regulation is pivotal for host bone metabolism.

Emerging evidence suggests that fermentable fibers, particularly those by the GM, significantly improve calcium absorption efficiency and bone metabolism.^24,118^ This is supported by human studies in children, where dietary fiber intake positively correlates with bone mineralization indices.^119^ Furthermore, fiber may mitigate environmental osteoporosis risks: NHANES data reveal an inverse association between fiber intake and osteoporosis risk in lead-exposed populations.^120^ Mechanistically, animal studies demonstrate that fiber supplementation improves bone microarchitecture via enhanced intestinal SCFAs production, restored epithelial barrier function, and promoted regulatory T cell (Treg) expansion—all hallmarks of gut–bone axis activation.^120^

Notably, gender-specific effects exist. Korean cohort studies demonstrated that fiber intake was protective for lumbar spine BMD in men but not in women, potentially due to SCFAs-mediated calcium transport and fiber-induced luminal distension.^121^ Although total carbohydrate intake shows conflicting associations with bone health, a high carbohydrate-to-fiber ratio is consistently linked to lower BMD and greater osteoporosis risk, particularly in postmenopausal women.^23,122^

In parallel, soluble fibers and indigestible carbohydrates—such as inulin-type fructans—enhance calcium retention, increase calcium absorption (by 58% in young adults and 42% in postmenopausal women), and beneficially modulate GM composition towards SCFAs-producing genera like *Allobacterium* and *Bifidobacterium*.^123,124^ Overall, these findings underscore the skeletal benefits of dietary fiber, particularly fermentable and prebiotic forms, through enhancing calcium metabolism and mitigating both nutritional and environmental risks for osteoporosis.

### Polyphenols

Polyphenols, a diverse class of plant-derived secondary metabolites abundant in fruits, vegetables, and herbs, exert broad-spectrum biological activities, including antioxidant, anti-inflammatory, anti-carcinogenic, and antimicrobial effects.^125^ Critically, as exogenous antioxidants, they constitute a key cellular defense mechanism by reducing oxidative stress and neutralizing free radicals.^126^ Moreover, Diets rich in polyphenols associate with delayed aging processes and reduced risks of multiple chronic diseases such as cardiovascular disease, atherosclerosis, cancer, type 2 diabetes, neurodegenerative disorders, and osteoporosis.^127,128^ Beyond these direct effects, polyphenols profoundly modulate GM composition by selectively promoting beneficial SCFAs-producing taxa (e.g., *Faecalibacterium* and *Akkermansia*) while suppressing pathogenic or pro-inflammatory bacteria.^129–131^ These microbial shifts enhance intestinal barrier integrity, reduce systemic endotoxemia (e.g., LPS), and elevate production of bone-protective metabolites (e.g., butyrate and propionate)—thereby constituting core mechanisms of the gut–bone axis.

Notably, Certain polyphenols demonstrate osteoprotective properties. For example, curcumin—the primary bioactive compound in turmeric—inhibits estrogen deficiency-induced bone loss in PMOP models.^132^ Intriguingly, while purified curcumin monotherapy is effective, polysaccharide-rich curcumin extracts exhibit attenuated anti-osteoporotic potency; however, they demonstrate superior efficacy in mitigating osteolytic progression.^132^ This suggests that food matrix composition and synergistic interactions may critically influence curcumin’s bone-protective potential. Similarly, resveratrol—a polyphenol in grapes, wine, and berries—exerts beneficial skeletal effects. A 12-month randomized controlled trial demonstrated that its supplementation significantly increased femoral neck BMD and reduced hip fracture risk in female participants, likely via BMD-enhancing mechanisms.^133^

Other polyphenols also contribute to bone health. Ellagic acid, a potent antioxidant and anti-inflammatory molecule, effectively attenuates trabecular bone loss, reduces oxidative stress in osteoblasts, and activates SIRT1-dependent signaling pathways in OVX mouse models,^134^ collectively supporting its osteoprotective potential. Similarly, Zibibbo grape seed extract, rich in flavonoids and phenolic compounds, promotes osteoblast proliferation and contributes to bone remodeling by mitigating oxidative stress and inflammation.^135^ Furthermore, flavonoids—a major polyphenol subclass abundant in fruits and vegetables—correlate with greater BMD and lower osteoporosis risk in population-based studies; specifically, flavones and flavonols exhibit strong associations with bone preservation.^136,137^ Among specific polyphenols, puerarin, an isoflavone derived from Pueraria lobata, demonstrates systemic anti-osteoporotic effects in animal studies, enhancing bone strength and inhibiting osteoclastogenesis.^138^

Critically, polyphenols may also benefit bone health via the gut–bone axis. Prunes (dried plums), rich in polyphenols and dietary fiber, improve BMD in postmenopausal women, particularly in individuals with low baseline BMD and favorable GM profiles.^139,140^ These findings suggest a dual mechanism of action: direct antioxidant and bone-anabolic effects, combined with indirect modulation of the intestinal microbiota. Indeed, emerging evidence indicates that polyphenols can function as prebiotic-like agents, altering gut microbial composition, enhancing SCFAs production, and improving gut barrier integrity. Consequently, these changes may collectively contribute to reduced bone resorption and improved bone formation. Thus, the interplay between polyphenols, the gut microbiome, and skeletal homeostasis represents a promising therapeutic avenue for osteoporosis prevention and management.

### Folate

A vegetarian diet is typically rich in folate, a water-soluble B vitamin abundant in plant-based foods, particularly dark green leafy vegetables and citrus fruits.^141,142^ Notably, emerging evidence indicates that folate may play a protective role against osteoporosis. An analysis of NHANES data involving 2 297 participants (mean age: 63.7 years; 49.9% female) revealed a significant association between higher dietary folate intake and reduced osteoporosis risk in the general population (*P*-trend = 0.005). Moreover, this association was notably stronger among women (*P*-trend <0.001) and adults over 60 years (*P*-trend <0.001), suggesting potential age- and sex-specific benefits of folate for skeletal health.^143^ Further, a threshold analysis identified 475.5 μg/day as a critical cutoff for dietary folate intake in predicting osteoporosis risk, indicating that intake below this level may increase vulnerability to bone loss.^144^ Consistent with these findings, higher dietary folate intake has been positively associated with BMD, with the strongest correlations observed in individuals over 80 years of age.^145^

Additionally, experimental studies also support folate’s role in maintaining bone health. For instance, in a high-fat diet-induced mouse model of osteoporosis, folic acid supplementation mitigated bone loss by modulating the AMPK signaling pathway.^146^ This suggests a mechanistic link between folate intake and bone metabolism under metabolic stress.

### Carotenoids

Carotenoids, a diverse group of fat-soluble compounds found in plants, are broadly classified into carotenes (hydrocarbons) and xanthophylls (containing hydroxyl or other oxygenated functional groups). Although a typical varied diet provides over (or >) 100 distinct carotenoids, studies detect only ~20 in human blood and tissues.^147^ Owing to their hydrophobic nature, carotenoids must be incorporated into lipid-containing mixed micelles for intestinal absorption before transport within lipoproteins to various tissues.^148^ Among these, β-carotene has shown protective effects against osteoporosis and is being explored as a potential therapeutic agent for bone loss.^149^ Similarly, lutein may help maintain bone mass by modulating bone resorption and formation, suggesting utility in preventing disuse-induced osteoporosis.^150^

Consequently, growing evidence indicates that dietary total antioxidant capacity (TAC) is strongly associated with bone health, particularly in postmenopausal women. Lower dietary TAC has been linked to an increased risk of osteoporosis, whereas higher TAC is correlated with improved bone mass in both premenopausal and postmenopausal women.^151–153^ These findings collectively emphasize the importance of antioxidant-rich dietary interventions in the management of PMOP. Therefore, enhancing intake of dietary antioxidants—including carotenoids (β-carotene, lycopene, lutein), vitamins C and E, folic acid, zinc, and selenium—combined with lifestyle strategies like regular physical activity, represents a promising approach to preserving bone health in individuals with PMOP.^154–156^

### Bidirectional crosstalk between dietary nutrition and the gut microbiota

The relationship between diet and the GM is not unidirectional but rather a dynamic, bidirectional interplay that significantly influences host physiology, including bone metabolism. On the one hand, dietary nutrition directly shapes the composition, diversity, and functional output of the gut microbial community. On the other hand, the microbiota itself modulates the intestinal microenvironment, affecting the digestion, absorption, and metabolism of dietary nutrients. This reciprocal interaction forms a critical foundation for the diet–gut–bone axis.

The influence of diet on microbial ecology is well-established. Dietary components serve as the primary substrates for gut microbes, exerting selective pressure that defines community structure. Divergent dietary patterns promote distinct microbial signatures. For instance, Western diets, high in saturated fats and refined sugars but low in fiber, reduce microbial diversity and enrich pro-inflammatory taxa (e.g., *Proteobacteria*) while depleting beneficial SCFA-producers.^21^ In contrast, Mediterranean diet and plant-rich diets abundant in fermentable fibers and polyphenols stimulate the growth of SCFA-producing genera such as *Faecalibacterium*, *Roseburia*, and *Prevotella*.^77,157,158^ These diet-induced shifts in microbial composition subsequently determine the metabolic landscape of the gut, including the production of key metabolites like SCFAs, TMAO, and various vitamins.

Conversely, the GM acts as a key modulator of diet nutrient bioavailability. It profoundly influences the host’s nutritional status by several mechanisms modulating nutrient absorption and metabolism within the intestinal lumen. First, microbial fermentation of dietary fibers to SCFAs (particularly butyrate, propionate, and acetate) lowers luminal pH, which enhances the solubility and absorption of minerals such as calcium and magnesium.^118,158,159^ This mechanism is a key pathway through which high-fiber diets exert bone-protective effects. Second, specific probiotic strains (e.g., *Lactobacillus* spp. And *Bifidobacterium* spp.) improve epithelial barrier function, thereby increasing transcellular and paracellular calcium uptake.^109,160^ Third, gut microbes participate in the metabolism of dietary compounds that are otherwise indigestible by host enzymes. For example, polyphenols are metabolized by microbial enzymes into more bioavailable metabolites, which then exert systemic anti-inflammatory and antioxidant effects relevant to bone health.^125,161,162^ Additionally, by regulating bile acid metabolism, the microbiota influences the absorption of fat-soluble nutrients including carotenoids.^163,164^ Certain commensals, notably some Bifidobacterium species, can synthesize folate de novo, directly supplying this essential vitamin to the host.^165,166^

This bidirectional crosstalk creates a feedback loop with direct implications for bone health. A dysbiotic microbiota, often induced by a low-quality diet, not only reduces beneficial metabolite production but can also compromise intestinal barrier integrity. This leads to increased systemic inflammation and impaired mineral absorption—processes detrimental to skeletal integrity. Conversely, a diet rich in diverse fibers and polyphenols fosters a eubiotic microbiota that enhances mineral bioavailability, reduces inflammation, and strengthens the gut barrier, collectively supporting skeletal homeostasis. This interdependence underscores why nutritional interventions aimed at bone health must consider their impact on the microbiota, and why microbiota-targeted therapies often rely on dietary context for efficacy.

In conclusion, the gut-bone axis is governed by a bidirectional dialogue in which dietary patterns sculpt the GM, and the microbiota in turn regulates host nutrition and metabolism through key molecular mediators such as SCFAs, polyphenols, and micronutrients. Since these bioactive compounds are not consumed or produced in isolation but are fundamentally shaped by the overall dietary pattern, a holistic perspective is essential to fully elucidate their role in bone metabolism and to develop effective nutritional strategies that support both the host and its microbial symbionts for optimal bone health.

## Dietary interventions targeting the gut–bone axis

**Fig. 2:**
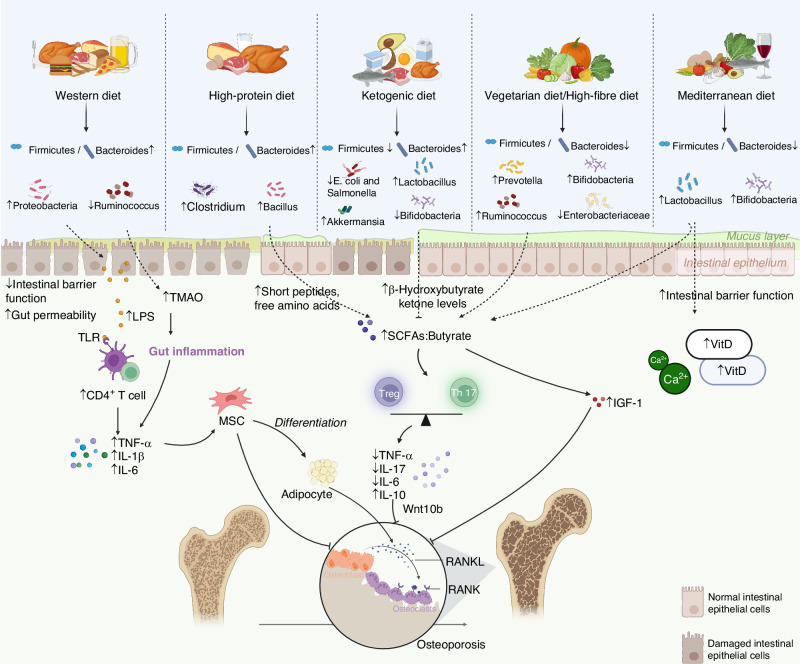
Impact of dietary patterns on the gut-bone axis. This schematic illustrates the critical role of dietary patterns in modulating the gut microbiota (GM) to influence bone metabolism along the gut–bone axis in the context of osteoporosis management. Western and ketogenic diets promote gut dysbiosis, increase intestinal permeability, and elevate systemic inflammation, thereby accelerating bone resorption and compromising intestinal and bone health. In contrast, Mediterranean, vegetarian, high-fiber, and high-protein diets generally foster a eubiotic GM enriched with SCFA producers, enhance intestinal barrier integrity, promote anti-inflammatory signaling, and stimulate bone formation, thus helping to maintain bone health and prevent osteoporosis. SCFAs short chain fatty acids, TMAO trimethylamine N-oxide, LPS lipopolysaccharide, TNF-α tumor necrosis factor-α, IL-10 interleukin-10, IL-1β interleukin-1β, IL-17 interleukin-17, IL-6 interleukin-6, vit D vitamin D, IGF-1 Insulin Like Growth Factor 1, RANKL receptor activator of nuclear factor-κB ligand, RANK receptor activator of nuclear factor-κB, Wnt10b Wnt Family Member 10B. Created with BioRender.com

As the primary environmental modulator of the GM, dietary patterns critically influence bone homeostasis through the gut–bone axis. Different regimens—from the Western and ketogenic diets to Mediterranean, vegetarian, high-fiber, and high-protein patterns—exert divergent effects on skeletal health, largely by promoting either a state of dysbiosis and inflammation or one of eubiosis and barrier integrity (Fig. 2). Building on this mechanistic foundation, the following sections critically evaluate how these specific dietary patterns differentially modulate the gut–bone axis and influence osteoporosis risk.

### Western diet

The Western diet is marked by a distinct food profile: high in red and processed meats, fried foods, high-fat dairy products, refined grains, and sugar-sweetened beverages, and low in fruits, vegetables, and dietary fiber. As such, it is commonly classified as a diet that is high in fat and sugar and low in fiber.^167^ This dietary pattern increases the risk of chronic diseases, including osteoporosis.^168^ Notably, high-fat diets (HFDs), a key component of this pattern, are established modifiable risk factors for osteoporosis.^44^

Western dietary pattern directly induces gut microbial dysbiosis. High fat and sugar content provides substrates that favor the expansion of pro-inflammatory taxa. The Western diet reduces microbial diversity and enriches pro-inflammatory taxa such as *Proteobacteria* while depleting beneficial SCFA-producing bacteria.^21^ The Western diet, characterized by high caloric and protein intake along with low carbohydrate and dietary fiber content, promotes the abundance of intestinal *Firmicutes*, *Clostridium*, *Acetitomaculum*, and *Faecalibacterium*, while inhibiting the prevalence of *Bacteroide*s.^156,169^ This shift reduces the production of SCFAs, particularly propionate and butyrate, and elevates LPS levels, thereby compromising bone health.^44^ Low dietary fiber intake leads to reduced levels of GM-derived SCFAs (e.g., acetate, propionate, and butyrate).^170^ These SCFAs are essential for maintaining intestinal barrier integrity and suppressing osteoclastogenesis via immune response modulation. In terms of dairy, milk and yogurt intake modulates the composition of the GM. One taxon assigned to the genus *Ruminococcaceae UCG-010 (ASV_24334)* exhibits an inverse correlation with milk consumption, whereas evidence supporting a positive association between another taxon belonging to the genus *Bifidobacterium (ASV_5993)* and milk intake remains weak. The detection rate of *Ruminococcaceae UCG-010* is significantly lower in milk consumers than in non-consumers. By contrast, Bifidobacterium is detected at a markedly higher rate in milk-consuming individuals relative to non-consumers. Compared with non-yogurt consumers, a taxon classified as the genus *Streptococcus (ASV_26229)* is positively correlated with yogurt intake, with its detection rate being significantly elevated in yogurt consumers. Furthermore, a taxon affiliated with the genus *Eisenbergiella (ASV_27085)* shows a negative association with cheese consumption,^171^ suggesting a possible microbiota-mediated effect.

The dysbiosis and reduced SCFA levels induced by the Western diet compromise intestinal barrier integrity, leading to increased translocation of bacterial endotoxins like LPS. Elevated circulating LPS triggers chronic low-grade inflammation, thereby affecting bone metabolism. Murine models of type 2 diabetes show that HFDs induce osteoporotic changes in trabecular bone within four weeks by suppressing osteogenesis, elevating bone resorption, increasing TNF-α expression, and promoting macrophage infiltration.^172^ These abnormalities coincide with disrupted femoral microarchitecture, altered bone turnover markers, and gut microbial dysbiosis.^173,174^ Similarly in humans, Western diets correlate with trabecular deterioration, especially in older adults.^175^ Epidemiological evidence also links traditional Western dietary habits to reduced BMD.^176,177^ Low dietary total antioxidant capacity is associated with elevated osteoporosis risk in postmenopausal women.^151^

Saturated fats, iron, and omega-6 polyunsaturated fatty acids (PUFAs) abundant in Western diets exacerbate bone loss through oxidative stress and inflammatory pathways.^178–180^ Excessive refined carbohydrates and added sugars amplify gut–bone axis dysfunction: high carbohydrate intake elevates fracture risk in postmenopausal women,^23^ high-sugar diets reduce bone strength in animals,^181^ hyperglycemia impairs osteoblast proliferation in vitro,^182^ and glucose ingestion increases urinary calcium excretion.^183,184^ High-fructose diets impair intestinal calcium transport.^185^ Sugar-sweetened beverages are strongly associated with lower BMD, with glucose-sweetened beverages exerting a more pronounced negative impact on calcium retention than fructose-sweetened ones.^186,187^ Although fructose may increase osteoblast numbers in rodents,^188^ its net effect on human bone remains unclear. Rodent studies indicate milk deprivation impairs bone quality,^189–191^ whereas human data show inconsistent associations between dairy consumption and BMD.^192^ Prebiotics such as lactulose and inulin can counteract some negative effects by enhancing gut barrier integrity, reducing osteoclast activity, increasing IL-10 levels, and restoring SCFAs production, leading to improved bone metabolism.^193,194^ Therefore, Western dietary patterns drive gut dysbiosis and bone deterioration, implying that interventions targeting the microbiota (e.g., fiber, prebiotics, dietary modification) may mitigate diet-induced osteoporosis risk.^122,195,196^

### Mediterranean diet

The Mediterranean diet is a well-recognized dietary pattern with favorable implications for skeletal health, featuring high intake of vegetables, fruits, fish, seafood, legumes, and nuts, moderate consumption of white meats, poultry, dairy products, and eggs, and limited intake of red wine.^197^ Olive oil serves as the primary source of dietary fat, contributing to a pattern rich in plant-based foods and monounsaturated fatty acids (MUFAs), with total fat typically accounting for ~30%–40% of daily energy intake.^198,199^ This dietary pattern is characterized by a favorable substitution of saturated animal fats with unsaturated vegetable oils, particularly those high in MUFAs.

The Mediterranean diet pattern exerts a significant regulatory effect on GM composition by selecting for beneficial microbial taxa. It is strongly associated with an increased abundance of butyrate-producing bacteria, including Genus *Oscillospira* (specifically, *Flavonifractor plautii*).^200,201^ A recent Spanish study further confirmed that individuals adhering to the Mediterranean diet harbor higher levels of various beneficial gut microbes, with the enrichment of *Bifidobacterium animalis* being particularly notable.^200^ This shift in microbial structure is attributed to the diet’s high content of dietary fiber and polyphenols, which serve as selective fermentation substrates. These compounds promote the proliferation of butyrate-producing bacteria and *Bifidobacterium* while simultaneously inhibiting the growth of pathogenic bacteria. In addition, adherence to the Mediterranean diet increases the *Prevotella*-to-*Bacteroides* ratio and microbial diversity.^74,169^

The GM alterations induced by the Mediterranean diet positively influence BMD through several interrelated mechanisms. Among the SCFAs, butyrate acts as a key mediator, activating regulatory pathways essential for the anabolic actions of parathyroid hormone in bone.^202^ Furthermore, butyrate-producing gut bacteria appear to enhance the bioavailability of vitamin D. Studies indicate that individuals colonized with these bacteria exhibit relatively higher levels of calcitriol, the active vitamin D metabolite.^203^ Concurrently, the increased abundance of *Bifidobacterium* contributes to improved BMD by reducing the production of cytokines that regulate bone resorption.^204^ These microbiota-mediated effects, combined with the direct osteoprotective actions of bioactive dietary components, work synergistically to attenuate bone loss and maintain skeletal health. By enhancing microbial diversity and promoting SCFAs production, these components improve gut barrier integrity, reduce systemic inflammation, and enhance bone remodeling—central mechanisms of the gut–bone axis.^205,206^ Epidemiological evidence supports these benefits, demonstrating that higher Mediterranean diet Index scores correlate with increased BMD and reduced fracture risk.^207–209^ Furthermore, lifestyle interventions combining energy-restricted Mediterranean diet with physical activity mitigate age- and weight-related BMD loss in older women with metabolic syndrome,^210^ while adherence to this diet associates with improved survival after vertebral fractures.^211^

The osteoprotective effects of the Mediterranean diet are partially mediated by its dietary fat profile. Specifically, the high consumption of MUFAs—with extra virgin olive oil as a key source—shows a positive correlation with increased BMD across multiple skeletal sites.^212,213^ MUFAs and a balanced omega-6 to omega-3 PUFA ratio modulate gut microbial ecology, leading to a reduced *Firmicutes/Bacteroidetes* ratio. This modulation enhances SCFAs production and restores Th17/Treg balance—two key mechanisms closely tied to bone homeostasis via the gut–bone axis.^72,88,214,215^ Further supporting the significance of intake level, Fang et al. demonstrated that only higher MUFA intake (>20.5 g/d) conferred a significant BMD benefit, underscoring the importance of both quantity and quality.^216^ PUFAs, particularly omega-3 fatty acids (EPA/DHA) from oily fish, improve femoral neck BMD in women.^217^ Their osteoprotection involves anti-inflammatory pathways and microbial modulation: Omega-3 intake enriches SCFAs-producing bacteria, enhances mucosal immunity, and suppresses gut-derived inflammation, collectively inhibiting osteoclastogenesis. Conversely, high arachidonic acid (omega-6 PUFA) intake without adequate EPA/DHA may accelerate bone loss.^217,218^ Randomized trials confirm that omega-3 supplementation combined with exercise improves BMD in postmenopausal women.^217^ Furthermore, higher omega-6/omega-3 ratios may be linked to lower BMD and increased fracture risk.^219^ Both α-linolenic acid and fish oil-derived EPA/DHA regulate microbiota and exert SCFA-mediated osteoprotection in human and animal models.^218,220–222^

In summary, adherence to the Mediterranean diet—rich in monounsaturated fats from olive oil, with a balanced omega-6/omega-3 ratio, and abundant in plant-based foods, fish, and seafood—is consistently associated with improved BMD and reduced fracture risk, particularly in vulnerable populations. This association is mediated through GM modulation, driven by the diet’s anti-inflammatory, antioxidant, and nutrient- rich components, which collectively support bone metabolism and mitigate age-related bone loss.

### Vegetarian diet

The vegetarian diet is a class of dietary patterns predominantly composed of plant-based foods, with key components including vegetables, fruits, nuts, seeds, whole grains, legumes, and cereals. It can be further categorized into distinct subtypes based on the extent of animal product exclusion: lacto-ovo-vegetarian (incorporating dairy and eggs), lacto-vegetarian (including dairy only), and vegan (excluding all animal-derived products). A notable nutritional feature of these vegetarian dietary patterns is their typically low content of saturated fats and cholesterol, coupled with a rich content of dietary fiber, polyphenols, folate, and carotenoids.^223,224^ Well-planned vegetarian diets are nutritionally adequate and safe for all life stages and confer health benefits including reduced blood pressure and lower serum cholesterol.^225,226^

The specific nutrient profile of the vegetarian diet shapes a distinct gut microbial ecosystem. It fosters a microenvironment conducive to the proliferation of anaerobic bacteria such as *Bacteroides*, *Roseburia*, *Eubacterium rectale*, *Ruminococcus bromii*, *Streptococcus*, and *Lactobacillus*.^76,227,228^ The abundant fermentable fibers and polyphenols serve as primary substrates for these microbes, driving increased production of SCFAs like acetate, propionate, and butyrate.^229,230^ Furthermore, vegetarian diets can modulate microbial composition by increasing the abundance of genera such as *Prevotella* and *Xylanibacter*.^95,231^ A specific mechanism involves the increased abundance of *Lactobacillus*, which enhances the production of tryptophan metabolites, notably indole-3-acetic acid (IAA).^232^

The vegetarian diet-mediated shifts in GM composition and metabolites exert anti-osteoporotic effects through multiple interconnected mechanisms that converge on bone metabolism. Primarily, the elevated levels of SCFAs (e.g., acetate, propionate, butyrate) alleviate systemic inflammation and reduce gut-derived LPS levels, collectively creating an anti-inflammatory milieu that suppresses osteoclast differentiation and activity.^233,234^ Concurrently, specific microbial metabolites, such as the tryptophan-derived indole-3-carboxaldehyde, activate the intestinal aryl hydrocarbon receptor (AhR). This activation stimulates the AhR/IL-10/Wnt signaling pathway, thereby restoring and enhancing intestinal barrier function and reducing systemic immune activation.^235^ Furthermore, supplementation with IAA and indole-3-propionic acid enhances M2 macrophages to secrete substantial levels of IL-10.^87^ IL-10 acts from the intestinal lamina propria to influence the bone marrow, concurrently promoting osteoblastogenesis and inhibiting osteoclastogenesis both in vivo and in vitro.^87,236^ Beyond these GM-centric pathways, the direct nutritional components of a plant-rich diet contribute significantly. The high intake of fruits and vegetables provides potassium and magnesium, which reduce the dietary acid load, thereby inhibiting osteoclastic bone resorption and promoting osteoblastic formation.^237^ Additionally, dietary antioxidants (e.g., vitamin C, vitamin E, carotenoids) directly counteract oxidative stress, a significant contributor to osteoporosis.^237–239^ The collective anti-inflammatory nature of a well-planned vegetarian diet thus counters pro-inflammatory diets that are associated with lower BMD and higher osteopenia risk.^240–242^ Thus, the vegetarian diet influences bone density through a convergent axis involving GM-derived anti-inflammatory metabolites, enhanced barrier integrity, systemic immune modulation, and direct nutritional action on bone cells.

While the aforementioned mechanisms reveal multiple pathways through which a vegetarian diet may exert bone-protective effects via the gut-bone axis, observational studies have yielded inconsistent conclusions regarding its actual impact on osteoporosis and fracture risk. An analysis of the NHANES database indicates that sticking to a plant-based diet is positively associated with BMD. Nevertheless, individuals following vegetarian or vegan diets require careful nutritional planning—particularly regarding calcium and vitamin D intake—and should undergo regular bone health monitoring.^243^ Specifically within vegan diets, higher levels of vitamin K and folate may help offset potential adverse effects on skeletal health.^244^ Higher folate intake or serum levels have been significantly associated with improved BMD and reduced fracture risk.^245^ For example, a sub-cohort of the Framingham Heart Study found that diets rich in fruits, vegetables, and cereals were associated with higher BMD in elderly men, with similar, though less pronounced, trends observed in women.^195^ However, emerging evidence suggests that plant-based diets, particularly vegan diets or those followed for ≥10 years, may be associated with an increased risk of osteoporosis, especially at the lumbar spine.^246^ A two-year study involving 210 postmenopausal women (half vegan, half omnivore) reported comparable rates of bone loss between groups, despite a higher prevalence of vitamin D insufficiency among vegans.^247^ Consequently, supplementation with vitamins D and K may help mitigate the increased hip fracture risk observed in some vegetarian populations.^248–250^

Large cohort studies have further revealed the complex association between the quality of vegetarian diets and bone health. For example, a study involving over 3 000 Scottish women linked diets rich in fruits and vegetables to reduced bone resorption, while diets high in processed foods were associated with lower BMD.^251^ However, a systematic review of studies in women aged ≥ 45 years reported limited and inconsistent evidence regarding the impact of fruit and vegetable intake on bone turnover markers, BMD, or fracture risk, attributing this largely to study heterogeneity and potential bias,^252^ highlighting the need to address methodological limitations in future research.

Dark green leafy vegetables (e.g., kale, spinach) are notable plant-based calcium sources, though their bioavailability is limited by oxalates, which impair calcium absorption.^253^ Despite lower calcium bioavailability compared to dairy, consuming large quantities of these vegetables can help meet daily calcium requirements, especially among individuals avoiding milk.^254,255^ Calcium absorption rates from vegetables range from ~20% to 40%, with slightly higher bioavailability reported for Brassica vegetables.^256,257^ Intriguingly, long-term adherence to plant-based diets does not consistently reduce fracture risk: a 30-year cohort study of >70 000 postmenopausal American women found no significant association between plant-based dietary patterns (healthy or unhealthy) and hip fracture risk.^258^

In summary, a vegetarian diet may support bone health and inhibit bone loss by reshaping the GM, promoting the production of SCFAs and specific microbial metabolites, and acting through multiple mechanisms including anti-inflammatory effects, immune regulation, and the direct supply of nutrients to bone. However, clinical findings are inconsistent, and strict long-term practice may carry risks such as inadequate intake of calcium and vitamin D, along with alterations in GM. Therefore, careful nutritional planning and monitoring are crucial for sustaining these skeletal benefits.

### High-fiber diet

A high-fiber diet is characterized by an abundance of non-digestible carbohydrates, notably dietary fiber and resistant starch.^259^ These complex glycans function as microbiota-accessible carbohydrates (MACs) that selectively fuel saccharolytic communities, promote production of host-beneficial metabolites, and help limit compensatory degradation of host-derived mucin glycans when fermentable substrates are scarce.^260^

These MACs serve as primary energy sources for the GM, particularly fiber-degrading members of the phyla *Bacteroidetes* and *Firmicutes* (e.g., *Prevotella, Ruminococcus*). In human intervention settings, fiber-rich whole-food patterns can exert measurable prebiotic effects on gut community structure, including increased fecal *bifidobacteria* and *lactobacilli* after whole-grain wheat consumption.^261^ Chronic consumption of a high-fiber diet increases microbial diversity and promotes expansion of beneficial taxa such as *Bifidobacterium, Lactobacillus, and Ruminococcu*s, concomitant with elevated SCFAs production,^224^ which can modulate osteoporosis. Notably, short-term escalation of whole-food fiber intake (targeting ~40–50 g/day for 2 weeks) is sufficient to shift individual microbiomes toward MAC-degrading taxa (including *Bifidobacterium* and *Lactobacillus*).^262^ Beyond “high fiber” as a broad category, discrete fiber chemistries can drive predictable functional outputs: structurally defined type-IV resistant starches induce substrate-specific and dose-dependent shifts in microbiome composition and selectively direct propionate versus butyrate production.^263^

The shifts in microbial composition and metabolic activity induced by a high-fiber diet directly impact bone homeostasis, primarily through SCFAs. Butyrate, a key SCFA produced by enriched bacteria like *Ruminococcus*, exerts multiple anti-osteoclastic effects.^83,264^ Mechanistically, it inhibits activation of the NLRP3 inflammasome in bone marrow-derived macrophages, thereby attenuating bone resorption, and suppresses IL-1β-induced osteoclast differentiation. Moreover, butyrate disrupts podosome organization, reducing formation of dense podosomal belts (actin sealing zones) and impairing osteoclast bone-resorptive function, which helps preserve skeletal integrity.^83^ By contrast, prolonged low-fiber intake leads to an irreversible decline in and reduced SCFA production, potentially increasing osteoporosis risk.^265,266^ Therefore, adequate dietary fiber intake is essential for maintaining a gut microbiome whose composition and function are conducive to bone health.

### High-protein diet

A high-protein diet is characterized by an elevated intake of protein, typically significantly above the recommended dietary allowance. Adequate intake of high-quality protein is essential for bone matrix synthesis, supplying amino acids for collagen formation and stimulating insulin-like growth factor-1 (IGF-1) secretion, which promotes osteoblast function.^267^

High protein intake, particularly from animal sources, increases nitrogen availability for proteolytic gut taxa (e.g., *Bacteroides, Clostridium*), which can alter community composition and metabolite production.^268,269^ Mechanistically, greater delivery of undigested protein and amino acids to the colon favors proteolytic fermentation and can shift microbial metabolism away from carbohydrate fermentation, changing the balance of microbial products. Microbial proteolysis yields metabolites including ammonia, hydrogen sulfide and branched-chain fatty acids; whereas moderate production may participate in host metabolic regulation, excessive generation can create a deleterious gut microenvironment, induce low-grade inflammation, impair mineral absorption and adversely affect bone metabolism.^270,271^ Moreover, animal-protein-rich diets are frequently accompanied by higher saturated fat intake and greater provision of sulfur-containing amino acids, factors that may further perturb microbial balance and promote systemic chronic inflammation—thereby jeopardizing bone health via the gut-bone axis.^76,267,272^

The net effect of a high-protein diet on bone health through the gut-bone axis is complex and context-dependent. Epidemiological and interventional data indicate that higher dietary protein intake, particularly from dairy sources, is associated with greater BMD, improved bone microarchitecture, and enhanced bone strength when calcium and vitamin D intakes are sufficient.^273^ This likely represents the dominant positive direct nutritional effect. Several observational studies report that total and animal protein intake, especially dairy protein, correlate with higher spinal and whole-body BMD in older adults^274^ and with greater predicted failure load and stiffness of peripheral skeletal sites in postmenopausal women.^275^ Certain protein sources, such as whey protein hydrolysate, have attenuated bone loss in high-fat diet models, plausibly through combined osteogenic and antioxidant effects.^276^

However, the potential negative modulation via the GM cannot be ignored, especially with excessive or unbalanced long-term high-protein intake. Animal studies show that maternal high-protein feeding can epigenetically impair offspring bone mass, underscoring the importance of early nutritional exposures.^277^ Although systematic reviews observe that many asserted harms of high-protein diets lack robust evidence, current nutritional guidance favors a moderate, high-quality protein intake (~1.0–1.2 g/kg/d, with higher targets for older adults) combined with adequate calcium, vitamin D, dietary fiber and abundant fruits and vegetables to maximize skeletal protection, support beneficial microbiota and maintain metabolic homeostasis.^278,279^

### Ketogenic diet

The ketogenic diet is characterized by very low carbohydrate intake, high fat intake, and moderate protein intake. Its effects on skeletal health remain controversial.^280^ From the perspective of the gut–bone axis, ketogenic regimens represent a marked shift away from fermentable carbohydrate supply toward lipid- and protein-derived substrates, and this substrate remodeling is a primary driver of GM changes that may secondarily influence bone remodeling. The ketogenic diet substantially remodels the GM, typically increasing the abundance of *Akkermansia* and *Lactobacillus*, reducing *Bifidobacterium* and pathogenic taxa (e.g., *Escherichia coli* and *Shigella*), and altering the SCFA profile.^281^ This pattern is consistent with limited carbohydrate substrate for saccharolytic taxa (notably *Bifidobacterium*) and a higher bile acid load under high-fat intake that selects for bile-tolerant communities, with downstream changes in SCFA output and bile acid transformation.^281^ This microbial shift may partly explain reported benefits of the diet for weight control, metabolic health, and neuroprotection. In some studies, ketogenic feeding produces a sustained decline in *Bifidobacterium*, which in turn reduces Th17 cell levels and thereby influences bone metabolism.^282^ Ketogenic regimens that include substantial protein may more strongly decrease the relative abundance of *Firmicutes* and increase that of *Bacteroidetes*.^283^

These microbiota changes induced by the ketogenic diet can plausibly affect bone density through immune and barrier-linked remodeling. Reduced fiber fermentation and altered SCFAs may weaken epithelial support and shift cytokine balance toward osteoclast activation, while the reported reduction in Th17 cells may counteract osteoclastogenesis in some contexts, helping to explain inconsistent skeletal outcomes. *Lactobacillus* enrichment is often interpreted as barrier- and inflammation-modulating, which could be bone-sparing when it lowers endotoxin-driven inflammation, however, if prolonged carbohydrate restriction reduces SCFA supply and mineral absorption efficiency, this may exacerbate calcium loss and favor bone resorption.^18,284^ Very low carbohydrate consumption can increase metabolic acid load and urinary calcium excretion, and circulating ketone bodies may act directly on bone cells. Animal studies report that long-term ketogenic feeding is associated with reduced bone mass, impaired biomechanical properties, and in some cases, osteoporosis.^285^ Notably, metformin has been reported to attenuate ketogenic diet-induced trabecular bone loss, suggesting a potential therapeutic approach.^286^

Taken together, the net effect of ketogenic diet–induced microbiota alterations on bone homeostasis remains unresolved. Whether these changes are protective or deleterious likely depends on dietary duration, fatty acid composition, protein source, and host-specific factors. Therefore, although ketogenic diets have demonstrated value in the management of certain metabolic disorders, their long-term effects on skeletal health remain unclear and warrant caution.

### Other dietary patterns

Other relatively rare dietary patterns include East Asian diets, Intermittent diet and Energy-restricted diet. East Asian diets, represented by traditional eating patterns in China, Japan, and Korea, emphasize vegetables, fruits, legumes, whole grains, and seafood and are rich in dietary fiber, plant protein, unsaturated fats, vitamins, and minerals. These diets may benefit bone health by modulating the GM and by supplying prebiotic substrates and probiotic foods.^287^ Specifically, Eastern special dietary practices—tea—can exert varying effects on GM depending on the tea variety. Liu et al.^288^ reported that polyphenols derived from green, red, and dark teas increase the relative abundance of beneficial gut microbial taxa, including *Bifidobacterium* and *Akkermansia*. These polyphenols have been associated with a lower *Firmicutes* to *Bacteroidetes* ratio, a microbial shift that may be favorable for bone health. Moreover, epigallocatechin gallate, a major polyphenolic component of green tea, has been shown to increase the relative abundance of *Lactobacillus reuteri* and *Lactobacillus gasseri*.^289^ Importantly, these taxa are implicated in enhanced intestinal calcium absorption, maintenance of osteoblast Wnt10b messenger RNA levels, stimulation of osteoblast proliferation, and direct suppression of osteoclast activity, thereby supporting skeletal homeostasis.^290–292^ Collectively, these effects may increase BMD and bone mineral content (BMC) and thus have potential benefit in osteoporosis. Nevertheless, additional mechanistic studies are required.

Intermittent diet denotes periodic alternation between feeding and fasting. Available evidence indicates that intermittent fasting can alter GM composition and function, often increasing the abundance and diversity of potentially beneficial microbes and thereby influencing bone health.^293,294^ However, severe energy restriction that produces overall malnutrition, or insufficient intake of protein, calcium, or vitamin D, will harm bone.^295,296^ Therefore, intermittent fasting regimens should ensure that feeding windows provide nutritionally complete and adequate meals to avoid potential negative effects on skeletal health. At present, high quality human studies directly linking intermittent fasting induced microbial changes to osteoporosis are scarce.

Energy restricted diets reduce total energy intake and are commonly used for weight loss or metabolic improvement. Modest energy restriction may benefit skeletal health through weight reduction, improved metabolic status, and modulation of the GM.^297–300^ Intuitively, reduced body weight can lessen skeletal loading and thus lower fracture risk, but excessive energy restriction can produce inadequate protein intake and other nutrient deficiencies that are harmful to bone.^301^ Careful design of energy restricted interventions is therefore required to preserve nutrient adequacy and protect skeletal health.

## Microbial community - targeted interventions in osteoporosis

**Fig. 3:**
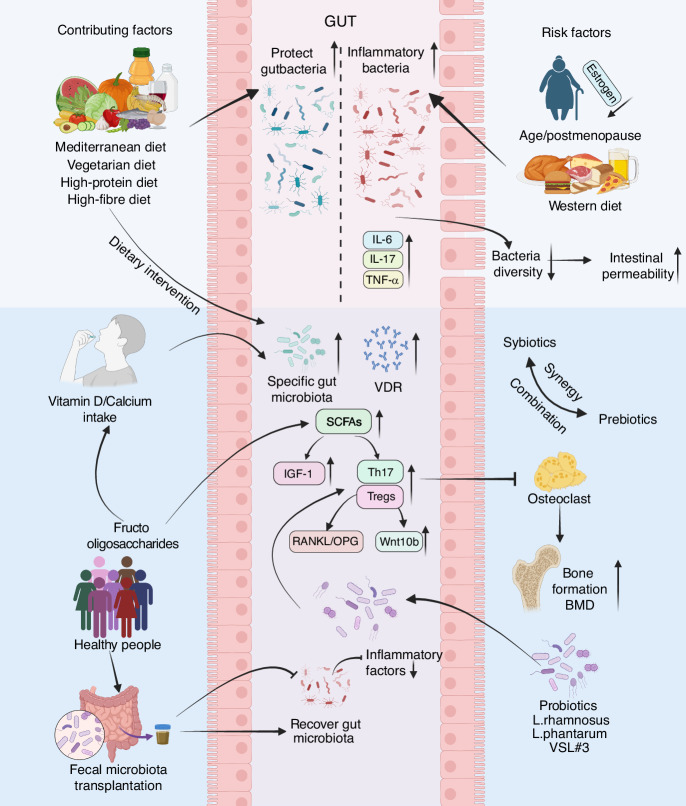
Microbiota-targeted strategies for osteoporosis. Schematic delineates how microbiota-targeted interventions (probiotics, prebiotics, synbiotics, fecal microbiota transplantation, and dietary modification) modulate the gut-immune-bone axis to prevent and treat osteoporosis by restoring microbial balance and counteracting dysbiosis induced by risk factors (e.g., aging, estrogen deficiency, poor diet). Th17 T helper 17 cells, Tregs regulatory T cells, OPG Osteoprotegerin, VDR vitamin D receptor, SCFAs short-chain fatty acids, TNF-α tumor necrosis factor-α, IL-17 interleukin-17, IL-6 interleukin-6, IGF-1 Insulin Like Growth Factor 1, RANKL receptor activator of nuclear factor-κB ligand, Wnt10b Wnt Family Member 10B, BMD bone mineral density. Created with BioRender.com

Targeting the GM has emerged as a promising therapeutic avenue for osteoporosis, moving beyond nutritional advice to direct and indirect modulation of the microbial community. As detailed in this section, interventions range from the administration of specific microbial strains or substrates (probiotics and prebiotics) and the transplantation of a complete microbial ecosystem (fecal microbiota transplantation) to the sustainable modification of dietary patterns. By targeting the gut-bone axis, these strategies operate through multiple interconnected mechanisms, including restoration of microbial diversity, enhancement of intestinal barrier function, modulation of immune responses, and regulation of key bone metabolic pathways (Fig. 3).

### Probiotics and prebiotics

Probiotics and prebiotics have emerged as promising adjunctive therapies for preventing and managing PMOP, increasingly employed as microecological agents in clinical settings. Specifically, recent studies have highlighted the beneficial effects of prebiotics, such as Fructooligosaccharides (FOS), on bone health. FOS-enriched diets significantly increased BMC, BMD, and trabecular volume by ~40% in vertebrae and 30% in the proximal tibia. Furthermore, FOS upregulates the expression of key osteoblast differentiation markers, indicating a direct stimulatory effect on osteoblast function.^302^ Mechanistically, the osteoprotective effects of FOS are primarily mediated by GM-dependent pathways. FOS supplementation increases the production and diversity of SCFAs, which helps maintain intestinal homeostasis and preserve beneficial microbial communities.^303^ Furthermore, FOS enhances the intestinal absorption of calcium^304^ and vitamin D.^305^ Adequate vitamin D levels, in turn, significantly increase the abundance of specific GM, thereby playing a role in the regulation of bone metabolism.^306^

Similarly, probiotic interventions—specifically those involving *Lactobacillus rhamnosus* and the multi-strain formulation VSL#3—have been demonstrated to preserve bone mass in animal models of estrogen-deficiency-induced osteoporosis and postmenopausal conditions.^290,307,308^ Mechanistically, probiotics such as *Lactobacillus* and *Bifidobacterium* maintain intestinal homeostasis, modulate immune responses, and enhance bone health by improving calcium absorption via both paracellular and transcellular pathways, thereby increasing calcium bioavailability.^89,204,309,310^ Specifically, these probiotics preserve gut epithelial integrity, modulate local and systemic immune responses (e.g., reducing TNF-α and IL-6 levels), and enhance intestinal calcium absorption through upregulation of TRPV6 and PMCA1b channels.^160,311^

Synbiotics (combinations of prebiotics and probiotics) exploit synergistic effects to optimize GM composition, enhance immunity, and support digestionn.^312^ For instance, Jia et al. developed a synbiotic milk powder (SMP) containing isomaltooligosaccharides, *Lactobacillus plantarum*, and calcium.^313^ In mice with prolonged calcium deficiency, SMP significantly improved apparent calcium absorption, increased serum calcium and phosphorus levels, enhanced BMD and trabecular number, and promoted osteoblast proliferation and differentiation,^313^ demonstrating its potential as a dietary intervention for calcium supplementation and bone remodeling.

Moreover, human studies further support these findings: Synbiotic supplementation in postmenopausal women (≥5 years post-menopause) has been shown to beneficially alter GM composition, enhance calcium and magnesium absorption, increase SCFA production, reduce intestinal pH, and improve fermentation of indigestible carbohydrates.^314^ Notably, in a study using female mice, a probiotic mixture containing *Lactobacillus paracasei* DSM13434, *L. plantarum* DSM15312, and DSM15313 (L. Mix) elevated the abundance of *L. paracasei* and *L. plantarum*, thereby leading to favorable shifts in microbiota composition and improved bone outcomes.^315^

Although calcium supplementation is often prescribed to address deficiencies, many individuals fail to meet recommended dietary calcium intake levels. Here, prebiotic fibers offer a viable alternative by enhancing calcium absorption and modulating the bone microenvironment. Furthermore, combining bioactive milk components with prebiotics or probiotics can amplify dairy’s bone-supportive effects, primarily through improved intestinal absorption of calcium and magnesium.^316^

Studies have shown that supplementation with *Lactobacillus reuteri* ATCC PTA 6475 prevents further deterioration of intestinal microbiota and reduces inflammatory status in elderly women with low BMD, thereby exerting a beneficial effect on bone metabolism.^317^ Similarly, a meta-analysis investigating probiotic supplements for osteoporosis management indicated their potential as a safe adjunct therapy to increase lumbar spine BMD in osteoporosis patients.^318^ Together, these studies provide robust evidence supporting the clinical translation and application of probiotics and prebiotics for bone health.

Collectively, probiotics and prebiotics enhance calcium metabolism and bone health through multiple mechanisms, including regulation of intestinal metabolites, maintenance of epithelial barrier integrity, and modulation of neural, immune, and endocrine signaling pathways.^319^ These findings highlight the potential of microbiota-targeted strategies as novel and complementary approaches to prevent and treat PMOP.

### Fecal microbiota transplantation

Probiotics, prebiotics, and fecal microbiota transplantation (FMT) have emerged as promising strategies for the prevention and treatment of PMOP by modulating the GM. Notably, FMT represents an innovative therapeutic approach, increasingly conceptualized as a novel form of “organ transplantation.” This procedure involves the isolation of beneficial GM from screened healthy human or animal donors via centrifugation and filtration, followed by their transplantation into the recipient’s gastrointestinal tract. The intervention aims to restore microbial balance, thereby improving intestinal function and ameliorating diseases associated with gut dysbiosis. Furthermore, FMT has demonstrated efficacy in modulating inflammation, enhancing immune responses, and regulating host metabolic pathways.^320,321^

Preclinical evidence strongly supports the role of the gut-bone axis in osteoporosis. Specifically, Yu et al. demonstrated that impaired barrier integrity, increased inflammation, and reduced microbial richness were present in the gut of OVX mice, and that microbiota depletion impeded osteogenic effects, which could be effectively improved by fecal transplantation from exercised mice.^60^ Similarly, transplantation of fecal microbiota from postmenopausal women with osteoporosis into mice resulted in significant bone deterioration.^322,323^ Critically, correlation analyses in these studies revealed that dominant microbial taxa from the donors were associated with altered bone metabolism and compromised intestinal permeability.^322,323^ Conversely, Ma et al. showed that therapeutic FMT not only restored gut microbial diversity in LPS-induced osteoporosis models but also upregulated the expression of long non-coding RNA TUG1, enhanced osteoblast activity, and improved bone microarchitecture.^324^ Additionally, Rui et al. provided further mechanistic evidence, demonstrating that oral FMT administration inhibited excessive osteoclastogenesis and prevented bone loss in OVX mice.^19^ Specifically, the treatment enhanced the expression of tight junction proteins, suppressed pro-inflammatory cytokine (TNF-α and IL-1β) production, optimized gut microbial composition, and increased fecal SCFA concentrations. Collectively, these results highlight FMT’s potential to restore intestinal barrier integrity, modulate immune responses, and improve bone metabolism.

Building on this, targeted modulation of specific microbial populations—such as increasing the abundance of *Lactobacillus*, *Bifidobacterium*, or optimizing the *Firmicutes/Bacteroidetes* ratio—through probiotics or FMT has been shown to mitigate age-related chronic inflammation and oxidative stress in musculoskeletal tissues. Therefore, these findings offer a strong rationale for microbiota-based interventions, particularly FMT, as a novel and complementary strategy for osteoporosis prevention and treatment.^320,325^

### Dietary intervention

Beyond direct microbial supplementation or transplantation, the sustainable modification of dietary patterns represents a foundational and accessible strategy for modulating the GM to prevent osteoporosis. Accumulating evidence indicates that long-term dietary habits exert profound and persistent effects on GM composition and function, thereby influencing bone metabolism through microbial-derived metabolites, immune regulation, and intestinal barrier integrity along the gut-bone axis.^36,37,92^

Healthy dietary patterns, such as the Mediterranean diet and the Dietary Approaches to Stop Hypertension (DASH), have been consistently associated with improved skeletal outcomes. Adherence to Mediterranean diet is linked to a reduced risk of hip fracture,^326^ while the DASH pattern correlates with higher lumbar spine BMD and a lower risk of osteoporosis, particularly among postmenopausal women.^327,328^ These benefits are mediated, in part, through GM modulation: Mediterranean diet and other plant-rich, high-fiber diets enrich SCFA-producing bacteria (e.g., *Faecalibacterium*, *Prevotella*), elevate the *Prevotella*-to-*Bacteroides* ratio and microbial diversity, and enhance gut barrier integrity.^74,75^ Consequently, systemic inflammation is attenuated (e.g., lower TNF-α, IL-6 levels), creating an environment that favors osteoblast activity and inhibits osteoclastogenesis^104–106^ The high content of polyphenols and MUFAs in such diets further contributes to an anti-inflammatory and antioxidant milieu conducive to bone preservation.^7,212,213^

Conversely, the Western-style diet—high in saturated fats, refined sugars, and processed foods—promotes gut dysbiosis. This dysbiosis is characterized by an increase in pro-inflammatory taxa (e.g., Proteobacteria) and a decrease in SCFA-producing bacteria.^21,56^ Such a pattern compromises intestinal barrier integrity, elevates circulating levels of LPS and TMAO, and ultimately accelerates bone resorption.^93,99^ Therefore, shifting toward more balanced, fiber-rich, and anti-inflammatory dietary patterns is a critical public health measure for skeletal health. A scoping review concluded that healthy dietary patterns emphasizing fruits, vegetables, whole grains, nuts, legumes, and low-fat dairy, while minimizing sugar-sweetened beverages, fried foods, and refined grains, are generally beneficial for bone health.^177^ Furthermore, the recent review emphasizes that a balanced diet providing adequate calcium, high-quality protein (especially from dairy), and abundant fruits and vegetables constitutes a cornerstone for the prevention of osteoporosis and fragility fractures.^273^

Individualized nutritional approaches are emerging as a research priority. For patients with chronic kidney disease, who face an elevated risk of fracture and sarcopenia, plant-dominant diets may help lower serum phosphorus and uremic toxin production, potentially preserving bone and muscle mass in non-dialysis patients, whereas higher protein intake may be necessary for patients receiving dialysis to mitigate bone loss and muscle wasting.^329^ Moreover, gene-diet interactions suggest that genetic background can modulate individual responses to diet; for instance, a genome-wide association study in older Hispanic adults identified significant interactions between genetic risk for osteoporosis and measures of dietary quality, including the DASH score and intake of sugar-sweetened beverages, implying that personalized dietary recommendations informed by genetic profiles could support early osteoporosis prevention efforts.^330^

In clinical practice, dietary counseling should emphasize dietary patterns that support both GM eubiosis and bone health.^331^ These include a high intake of vegetables, fruits, legumes, whole grains, nuts, and fish; adequate calcium and high-quality protein; and limited consumption of red meat, processed foods, and sugar-sweetened beverages. Such a diet establishes an ecological niche that favors beneficial commensal bacteria in the gut, thereby supporting skeletal health and helping to prevent osteoporosis.^28^ Future strategies may integrate dietary guidance with microbiome profiling to develop personalized nutrition plans for osteoporosis management.

In summary, dietary intervention is a fundamental component of the microbiota-targeted approach to osteoporosis. It offers a scalable, sustainable, and economically feasible means to modulate the GM, with the potential to attenuate bone loss and reduce fracture risk, particularly in high-risk populations such as postmenopausal women.

### Comparative strategies for osteoporosis management

**Fig. 4:**
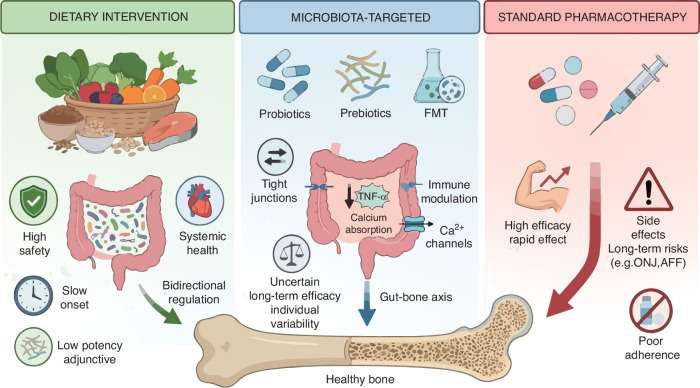
Comparative advantages and limitations of dietary interventions, gut microbiome–targeted strategies, and standard pharmacotherapy in osteoporosis management. The schematic compares three osteoporosis management strategies converging on improved bone health. Dietary intervention is safe but slow-acting, relying on microbiome support. Microbiota-targeted therapies modulate the gut-bone axis by reinforcing tight junctions, suppressing TNF-α-driven inflammation, and enhancing intestinal Ca²⁺ transport. Standard pharmacotherapy provides faster, higher efficacy through direct antiresorptive/anabolic actions but carries a risk of adverse events, supporting individualized strategy selection. ONJ osteonecrosis of the jaw, AFF atypical femoral fracture, FMT fecal microbiota transplantation, TNF-α tumor necrosis factor-alpha, Ca^2+^ calcium ion

The effective management of osteoporosis necessitates a strategic integration of interventions that target both the long-term modification of bone health trajectory and the immediate prevention of fragility fractures. This comparative analysis evaluates the distinct roles, advantages, and limitations of foundational strategies, namely dietary interventions and novel gut microbiome–targeted approaches, against standard pharmacotherapy (Fig. 4). The objective is to delineate their complementary positions within a cohesive clinical framework, underscoring the rationale for a sequential and individualized treatment paradigm.

Beginning with the most foundational level of intervention, dietary modification represents the cornerstone of long-term skeletal health management. Dietary patterns such as the Mediterranean diet and DASH-style eating provide a low-risk, system-wide foundation that can support skeletal health but generally act gradually and depend heavily on sustained adherence, with effect sizes that are typically modest at the individual level.^332^ As outlined above, diet-based care offers broad cardiometabolic and anti-inflammatory co-benefits and can be sustained long term with minimal toxicity, yet its slow kinetics and adherence dependence limit its ability to address imminent fracture risk as a stand-alone strategy. Consequently, dietary interventions are best viewed as a foundational, long-term component of care, which can be strategically augmented by more targeted approaches.

One such targeted intervention arises from recognizing that diet profoundly shapes the gut microbiome, a key regulator of bone metabolism. Microbiome-focused approaches thus extend the principles of lifestyle care by directly targeting the gut-bone axis through improved barrier function, inflammatory tone, and mineral handling. Probiotics, prebiotics, and synbiotics are comparatively scalable and low-risk options that may enhance intestinal Ca^2+^ transport and dampen TNF-α/IL-6–associated inflammation, whereas FMT may more profoundly “reset” dysbiosis but introduces higher procedural complexity and greater uncertainty in standardization. However, clinical translation remains constrained by heterogeneity in strains/formulations, limited high-quality outcomes data, and additional safety and standardization concerns for more invasive modalities such as FMT.^299^ Therefore, while representing a promising mechanistically-driven adjunct, microbiome modulation shares the preventive and long-term orientation of its dietary foundations.

For patients at high or imminent risk of fracture, however, the slow timelines of lifestyle and microbiome interventions necessitate a shift to pharmacotherapy capable of delivering rapid risk reduction. Standard pharmacotherapies—including antiresorptive agents that directly suppress osteoclast-mediated bone resorption and anabolic therapies that stimulate osteoblast activity—can deliver rapid and clinically meaningful fracture-risk reduction in appropriately selected high-risk patients, often with larger effect sizes than non-pharmacological strategies.^333^ Nevertheless, these benefits must be balanced against potential risks, as pharmacotherapy requires structured monitoring and risk stratification due to rare yet serious adverse events such as medication-related osteonecrosis of the jaw and atypical femoral fractures.^333,334^ Moreover, real-world effectiveness is frequently limited by poor adherence and persistence, which may erode the expected fracture-risk reduction observed in clinical trials. Thus, while pharmacotherapy remains essential for immediate risk mitigation, its use highlights the need for individualized strategy selection based on patient risk profile, tolerability, and likelihood of adherence.

Given the complementary strengths and limitations of these diverse strategies, an integrated, sequential treatment paradigm is recommended. Collectively, these considerations justify a stepped, combined therapeutic strategy. Initial interventions should focus on diet and microbiome-directed approaches to reinforce upstream biology and ensure long-term safety. In contrast, pharmacotherapy is prioritized when rapid fracture-risk reduction is required. Integrating these modalities aims to maximize therapeutic efficacy while minimizing potential risks.

## Conclusions and prospectives

The gut-bone axis is increasingly recognized as a significant research focus with advancing insights into GM. This complex network links diet, the gut, and bone tissue through regulatory mechanisms spanning inflammatory responses and immune modulation. Although significant progress has been made in understanding how GM influences bone metabolism, critical knowledge gaps persist.

While numerous studies demonstrate that the gut microbial diversity and composition differ between osteoporosis patients and healthy individuals, variations in study populations, sequencing platforms, and dietary habits contributes to inconsistencies in reported microbiota profiles. For instance, some studies report reduced abundance of SCFA-producing bacteria (e.g., *Bacteroides*, *Prevotella*) in individuals with osteoporosis, whereas others highlight the enrichment of *Ruminococcus* and *Allisonella* in this population.^57,71,335^ These discrepancies highlight the substantial heterogeneity across study populations and methodologies.

However, the extent to which dietary patterns modulate the GM and their subsequent regulatory impacts on bone health remains incompletely understood. The Mediterranean diet—rich in polyphenols,^7,126^ MUFAs,^33,198^ and dietary fiber^24,120,121^—is associated with enhanced BMD and reduced fracture risk. Conversely, adherence to a Western diet is linked to dysbiosis of pro-inflammatory microbiota and reduced SCFA production, impairing the intestinal barrier and exacerbating systemic inflammation.^22,30,240^ Additionally, the precise associations between GM and other influencing factors (such as lifestyle and genetics) require further elucidation.

The clinical translation of GM-based interventions—including probiotics, prebiotics, and FMT—for osteoporosis management shows promise but is still nascent. Although animal studies demonstrate that specific probiotics (e.g., *Lactobacillus rhamnosus*, *Bacillus coagulans*) can enhance bone mass via immune modulation and RANKL pathway inhibition, human clinical trials remain scarce, and substantial heterogeneity exists in probiotic strains and dosages.^65,106^ Similarly, while FMT reverses ovariectomy-induced osteoporosis in models, further investigation is needed to address its safety, donor selection criteria, and procedural standardization.^19^ Furthermore, emerging research highlights that microbial metabolites—including SCFAs and TMAO—also play a pivotal role in bone remodeling dynamics.^42,99^ However, the dosage, source, and interindividual variability in microbial metabolic capacity during the clinical translation of these metabolites require further exploration. Collectively, these persistent gaps underscore a deeper, transversal challenge that impedes the entire field: the translation of gut microbiome research into reliable clinical applications.

Notably, the successful translation of such mechanistic insights into effective clinical interventions is hindered primarily by the lack of end-to-end standardization that spans upstream manufacturing through clinical application. The high heterogeneity observed across probiotic and prebiotic studies reflects the absence of unified standards for critical steps such as strain identification, fermentation processes, and formulation definition. Consequently, the evidence base is fragmented and poorly reproducible, which impedes the formation of authoritative clinical guidelines. This standardization gap is particularly acute for complex living biological therapies, including FMT and other live biotherapeutic products. In the absence of widely accepted standards for donor screening, preparation procedures (for example centrifugation and cryopreservation), and dosing, interindividual variability in efficacy, unresolved long-term safety concerns, and potential immunogenicity remain major regulatory and ethical challenges. Even approaches that target microbial metabolites at the molecular level, such as butyrate, face classical pharmaceutical barriers, including rapid in vivo metabolism, difficulty maintaining therapeutically effective local concentrations, and the lack of specific delivery systems. Accordingly, translational research must move beyond superficial associative observations and establish a standardized scientific framework that covers the full life cycle of research and development, manufacturing, and clinical evaluation. Achieving this goal will require deep multidisciplinary integration of microbiology, pharmaceutical engineering, clinical medicine, and regulatory science, precise definition of critical quality attributes, and development of robust potency assays. These measures are essential to advance intestinal microbiome interventions from empirical exploration to a rigorous and standardized precision medicine paradigm.

Finally, estrogen signaling plays a key role in PMOP by modulating intestinal flora balance;^63^ nevertheless, the interplay between host genetics, estrogen signaling, and intestinal flora remains underexplored. Estrogen deficiency alters flora composition and increases intestinal permeability, contributing to immune dysregulation.^193^ Consequently, future research necessitates integrated experimental approaches to elucidate this complex relationship, which is imperative for advancing personalized osteoporosis therapies.

In summary, diet-microbiota interactions hold promise for osteoporosis prevention and management, yet future research priorities should include: (1) longitudinal, randomized human studies;(2) standardized microbiota analysis protocols; and (3) the development of personalized nutrition frameworks integrating genetic and microbial data. Such interdisciplinary approaches are essential to refine gut-directed therapeutic strategies for osteoporosis prevention, particularly in postmenopausal populations.

## Data Availability

No datasets were generated or analysed during the current study.
